# Optimization of Provitamin A Maize and Iron‐Rich Bean‐Based Composite Flour for Improved Child Nutrition

**DOI:** 10.1002/fsn3.71390

**Published:** 2025-12-26

**Authors:** Amos Asiimwe, Juliana Nambwayo, Boniface Brian Odong, Ivan Muzira Mukisa, Robert Fungo

**Affiliations:** ^1^ Department of Nutrition, Food Science and Technology Bugema University Kampala Uganda; ^2^ National Agricultural Research Laboratories‐Kawanda Kampala Uganda; ^3^ Department of Food Technology and Nutrition Makerere University Kampala Uganda; ^4^ KU Leuven Faculty of Science Leuven Flanders Belgium

**Keywords:** bio‐fortification, iron, optimization, provitamin A bio‐fortified maize, response surface methodology, vitamin A

## Abstract

Bio‐fortification is a strategy that has been proven to not only reduce the prevalence of micronutrient deficiencies, but also to tackle other forms of malnutrition. Therefore, the inclusion of flour from bio‐fortified crops as major components of composite flours can significantly alleviate malnutrition. In this study, the effect of replacing white maize with provitamin A bio‐fortified maize in a bean‐based composite flour was studied. Response surface methodology was used to optimize the nutrient composition and overall sensory acceptability of the composite flour. Design Expert 2018 (Version 12) was used to generate 30 treatments where provitamin A, iron, phytate concentration, and overall acceptability were considered as the response variables. Six locally available cereals and legumes were considered as independent variables and were included in the composite flour at different levels. Bio‐fortified maize was included at a level between 50% and 60%, high iron‐rich beans at 15%–20%, sesame at 5%–10%, soy at 10%–15%, wheat and sorghum at 5% each. An optimal formulation of the composite was achieved with 57.89% maize, 17.11% beans, 5% sesame flour, 10% soy bean flour, and 5% of each of the wheat and sorghum flour. The respective values for provitamin A, iron, phytate content, and overall acceptability for the optimal formulation were 1.58 μg/g RAE of beta carotene, 6.0 mg/100 g of iron, 54.20 mg/100 g of phytates, and overall acceptability at 7.10 respectively. All models predicting these values were satisfactory and had a nonsignificant lack of fit (*p* < 0.05). This indicated the suitability of each model in predicting the responses and optimizing the formulation for the production of a quality provitamin A maize‐iron bean‐based composite flour.

## Graphical abstract

In order to improve child nutrition, the study optimized a composite flour derived from iron‐rich beans and provitamin A maize using Response Surface Methodology. High levels of β‐carotene (1.58 μg/g), iron (6.0 mg/100 g), and sensory acceptability were obtained from the ideal blend (57.9% maize, 17.1% beans, 10% soy, 5% sesame, 5% wheat, and 5% sorghum), indicating that bio‐fortified crops can enhance the nutritional value and consumer appeal of cereal‐legume composite flours.

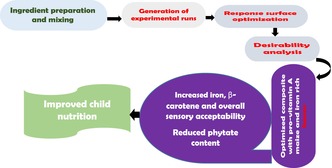

## 1 Introduction

Under nutrition and micronutrient deficiencies are prevalent in low‐ and middle‐income countries and contribute to under‐five morbidity and mortality (Mulualem 2015; Mutumba et al. 2023). Iron deficiency anemia and vitamin A deficiency are the most common and widespread micronutrient deficiencies afflicting more than half of the global human population (Harvest plus 2018; Mandial et al. 2023). Vitamin A deficiency (VAD) is the leading cause of preventable blindness in children and increases the risk of severe infections (Gautam et al. 2021). In Uganda, VAD affects 9% of children under the age of 5 years (Uganda Bureau of Statistics [UBOS] 2016). On the other hand, iron deficiency anemia (IDA) is the most common nutritional disorder affecting more than one‐third of the population worldwide and leading to microcytic anemia, fatigue, weakness, shortness of breath, and dizziness (Stevens et al. 2013). In Uganda, IDA affects 53% of children under the age of 5 years (UBOS 2016). Nondiversified cereal and plant‐based foods that dominate diets in the developing world are poor in micronutrients which further aggravates the risk of these micronutrient deficiencies (Mandial et al. 2023). There have been efforts to explore sustainable food‐based strategies such as bio‐fortification of staples to address the prevalent micronutrient disorders. Cereals and legumes like white maize and common beans were identified as primary targets for bio‐fortification because they are widely consumed (Singh et al. 2024) and thus increasing their micronutrient density could go a long way in reducing the prevalence of micronutrient deficiencies (Andersson 2022). Various food products including composite flours have been developed using common cereals and legumes (Akonor et al. 2017; Ndagire et al. 2015). However, there is a dearth of information on the development of optimized composite flours made from more than one bio‐fortified crop. The main objective of this study was therefore to optimize the nutrient composition of a provitamin A bio‐fortified maize and bio‐fortified bean‐based composite flour to improve the micronutrient status of children between 6 and 59 months.

## 2 Materials and Methods

### 2.1 Selection of Raw Materials

Local varieties of provitamin A bio‐fortified orange maize (Naro maize 63 vitA), high iron beans (Naro bean 1), soybeans, whole wheat, sorghum, and sesame were procured from markets in Kampala, Uganda. Orange maize flour was the primary component of the composite flour due to its high provitamin A content (Vigneshwaran et al. 2020); iron‐rich bean flour was included for its superior iron levels compared to common beans (Glahn et al. 2020). Sesame flour was added for its oil content, which aids in vitamin A absorption, and its nutty flavor offsets undesirable beany notes (Mostashari and Mousavi 2024; Pusadkar et al. 2015). Soybean flour was used to enhance the protein content, providing high‐quality plant‐based protein comparable to animal sources (Qin et al. 2022). Sorghum contributed to porridge color and provided essential minerals like potassium, phosphorus, and fiber (Abdelrahim et al. 2016; Khoddami et al. 2023). Whole wheat was included to boost the energy content of the composite flour (Shewry 2015).

### 2.2 Preparation of Flours

The flour from the provitamin A rich maize was prepared as described elsewhere by Margaret et al. (2017). The high iron bean flour was prepared in accordance with a method described by Byarugaba et al. (2023). Sesame flour was prepared as described by Makinde and Akinoso (2014). Soy flour was prepared in line with the method described by Udomkun et al. (2022). Whole wheat flour was prepared as described by Bhat et al. (2020) while sorghum flour was prepared according to a method described by Irondi et al. (2019).

Prior to preparation of the flours, the grains were manually sorted. The iron‐rich beans were then roasted at 200°C for 15 min, sesame seeds at 160°C for 25 min, soybeans at 200°C for 60 min, and sorghum at 135°C for 15 min in an oven (Infrared food oven GL‐2A; Guangzhou Itop Kitchen Equipment Co Ltd., Guangdong, China). The roasted ingredients, provitamin A rich maize and whole wheat, were individually milled into coarse flour using a wonder mill grinder (Wonder mill Pocatello, Idaho, USA). The ground material was manually sieved using a 0.6‐mm sieve. The resulting flour was packaged in polyethene bags awaiting further use.

Roasting of the iron‐rich beans, sesame seeds, soybeans, and sorghum grains was mainly done to reduce the anti‐nutritional properties of the raw grains (Vaishnavi and Mamta 2024; Zhou et al. 2024). In addition, roasting of sorghum was done to improve its functional properties such as water absorption capacity (Batariuc et al. 2023).

### 2.3 Experimental Design

Design Expert 2018 (Version 12) was used to design the experiment for the study. Response surface methodology, based on the D‐optimal mixture design, was adapted with six independent variables, namely: flours of orange maize, iron‐rich beans, sesame, soybean, sorghum, and wheat against three independent variables: provitamin A (beta carotene), iron content, phytate content, and overall acceptability. The independent variables and their levels of inclusion into the composite flour are summarized in Table 1. Based on the D‐optimal mixture design, a total of 30 treatment runs, as summarized in Table 2 were generated. The runs represent the various possible flour combinations for the composite flour. The desirability function approach was used in optimizing and selecting the best combination that produced the best quality of the optimized composite flour.

**TABLE 1:** Coded and actual percentage levels of the different components used in the generation of the experimental runs.

Component	Name	Minimum	Maximum	Coded low	Coded high
A	Orange maize	50	60	+0 ↔ 50	+1 ↔ 60
B	Iron‐rich beans	15	20	+0 ↔ 15	+0.5 ↔ 20
C	Sesame	5	10	+0 ↔ 5	+0.5 ↔ 10
D	Soybean	10	15	+0 ↔ 10	+0.5 ↔ 15
E	Sorghum	5	5	+0 ↔ 5	+0 ↔ 5
F	Wheat	5	5	+0 ↔ 5	+0 ↔ 5

**TABLE 2:** The D‐optimal experimental design for the different flour combinations.

Run	% Composition					
	A. Orange maize	B. Beans	C. Sesame	D. Soybean	E. Sorghum	F. Wheat
1	52.6	20.0	5.0	12.4	5.0	5.0
2	56.0	19.0	5.0	10.0	5.0	5.0
3	56.0	19.0	5.0	10.0	5.0	5.0
4	50.0	18.4	10.0	11.6	5.0	5.0
5	50.0	20.0	7.3	12.6	5.0	5.0
6	50.0	20.0	5.1	14.9	5.0	5.0
7	53.6	17.1	7.1	12.1	5.0	5.0
8	53.6	20.0	6.3	10.0	5.0	5.0
9	55.5	17.0	5.0	12.5	5.0	5.0
10	54.3	15.7	5.0	15.0	5.0	5.0
11	60.0	15.0	5.0	10.0	5.0	5.0
12	57.2	15.0	7.8	10.0	5.0	5.0
13	50.0	17.9	7.1	15.0	5.0	5.0
14	52.5	15.0	9.9	12.6	5.0	5.0
15	53.6	17.1	7.1	12.1	5.0	5.0
16	53.6	17.1	7.1	12.1	5.0	5.0
17	53.6	17.1	7.1	12.1	5.0	5.0
18	50.0	17.9	7.1	15.0	5.0	5.0
19	54.3	15.7	5.0	15.0	5.0	5.0
20	53.6	17.1	7.1	12.1	5.0	5.0
21	54.6	15.4	10.0	10.0	5.0	5.0
22	51.6	20.0	8.4	10.0	5.0	5.0
23	54.6	15.4	10.0	10.0	5.0	5.0
24	52.3	15.1	7.6	15.0	5.0	5.0
25	50.0	18.4	10.0	11.6	5.0	5.0
26	51.6	20.0	8.4	10.0	5.0	5.0
27	54.8	15.0	7.5	12.7	5.0	5.0
28	53.6	17.1	7.1	12.1	5.0	5.0
29	50.0	15.0	10.0	15.0	5.0	5.0
30	57.7	15.0	5.25	12.	5.00	5.0

### 2.4 Analyses

#### 2.4.1 Determination of the Phytate Content of the Flour

Phytic acid content was determined according to procedures described by Udomkun et al. (2022) using a colorimetric (Wade reagent) method. About 1 g of sample was weighed out and macerated in a mortar and pestle to extract phytic acid using 100 mL of 2.4 M HCl mixed with 0.1 M sodium chloride. The clear supernatant of filtrate of the extract was further diluted 10 times into a 100 mL volumetric flask and made to the mark using distilled water. Thereafter, 1 mL of clear solution was diluted 10 times with distilled water and collected for color development. About 3 mL of the diluted sample was mixed with 1 mL of Wade reagent (made by mixing 0.03% FeCl_3_·6H_2_O and 0.3% sulfosalicylic acid) and thoroughly vortexed and centrifuged for 10 min. A series of standards containing 0, 1.12, 2.24, 3.36, 5.6, 7.84, and 11.2 mg/mL was prepared from a sodium salt of phytic acid M.W. 660.04 g and used in generating a standard calibration curve. Absorbance of color reaction products for both samples and standards was read at 500 nm on a spectrophotometer (Spectroquant Pharo 300M, EU). The amount of phytate in the samples was calculated as shown in Equation (1) from calibration curve.

(1) $$y=1.3959x-0.0298\;R^{2}=0.91$$

where *y* is the concentration of the phytates in the sample and *x* is absorbance.

#### 2.4.2 Determination of Total Carotenoids in the Composite Flour

Total carotenoids content was determined according to the method of Schmalzer et al. (2008). Briefly, about 2 g of composite powder were ground with about 45 mL of cold acetone and about 3 g of celite using a mortar and pestle. The mixture was filtered (with glass wool placed into a funnel) into a 50 mL volumetric flask while adding acetone to the residue until it was white and made to the mark. This was then added to a separating funnel containing about 35 mL of petroleum ether. To the mixture in the separating funnel was then added 250 mL of distilled water and the lower layer removed by opening the tap at the lower end of the volumetric flask. This step was repeated four more times. About 10 g of anhydrous sodium sulfate was added onto the funnel with glass wool to form a bed. The separating funnel and the funnel with glass wool were rinsed with petroleum ether into a 50 mL volumetric flask to the mark. Absorbance was measured at 450 nm (Genesys 10‐UV spectrophotometer; Thermo Electron Corporation, Madison, WI, USA) against petroleum ether as a blank. The β‐carotene content was calculated using Equation (2) below:

(2) $$\beta ‐\text{Carotene}\;\left(\mathrm{\mu g}\;\mathrm{RAE}/100\;g\right)=\frac{A_{b}\times V\left(\mathrm{mL}\right)\times 10^{4}}{12A_{1\;\mathrm{cm}}^{1\%}\times p\left(g\right)}$$

where *A*_b_ = absorbance; *V* = total extract volume; *p* = sample weight; $A_{1\mathrm{cm}}^{1\%}$ = 2592 (β‐carotene extinction coefficient in petroleum ether). RAE = retinol activity equivalents; 1 retinol activity equivalent = 12 μg β‐carotene in food.

#### 2.4.3 Determination of the Iron Content of the Flour

The iron content of the flour was quantified according to the method described by Peruchi et al. (2014) using Energy Dispersive X‐Ray Fluorescence (EDXRF) spectrometer, model EDX‐720 (Shimadzu, Japan). Cuvettes each covered with a polypropylene film were used. Each flour sample was added into a separate cuvette until it was three quarters full. The cuvettes containing the samples were then loaded into the machine, and the machine was turned on and allowed to run until the process was complete. The concentration of iron in each sample was then read directly from a computer in ppm. The X‐ray tube used was rhodium, and the working atmosphere was nitrogen. The excitation energy used was 50 keV, and the detector operated at −176°C. Analyses were performed in triplicate.

### 2.5 Evaluation of the Overall Sensory Acceptability of the Flour

#### 2.5.1 Preparation of Porridges

Porridges were prepared as described by Onoja et al. (2014) where 100 g of flour from each of the composite samples was mixed with 100 mL of cold water to make a paste. After that, 500 mL of boiled hot water was added to the paste. The paste was cooked for 10 min while stirring occasionally.

#### 2.5.2 Sensory Evaluation of the Porridges

Overall consumer acceptability tests of the porridge from each of the 30 runs were carried out by a team of 30 untrained panelists who were mothers or caretakers of children using a 9‐point hedonic scale (1 = dislike extremely to 9 = like extremely). To avoid taster fatigue, each panelist was required to evaluate porridge from six runs only in each session. About 15 mL of each of the prepared porridge samples at about 45°C was presented to the panelists in labeled plastic containers. Each panelist was required to score the degree of overall acceptability of the porridges. Since the study did not involve collecting any personal data or biological samples from the panelists, institutional review was not sought; rather, individual consent to take part in the sensory evaluation of porridges was obtained. Each panelist was required to sign a written consent form before taking part in the sensory evaluation exercise.

### 2.6 Data Analysis

To optimize the composite mixture formulations, data were analyzed by Response Surface Methodology (RSM) procedures using Design Expert 2018 (Version 12) software. The lack of fit and coefficient of determination *R* ^2^ analyses were further done to test the adequacy of the developed models. Optimized conditions predicted by the software and experimental values of the response variables were compared using a *t*‐test (*p* < 0.05).

## 3 Results and Discussion

### 3.1 Provitamin A, Iron, Phytate, and Overall Acceptability of the Optimized Bio‐Fortified Maize and Bean Composite Flour

The provitamin A, iron, phytate, and overall acceptability varied from 1.00–1.61 μg RAE, 5.62–6.39 mg/100 g, 53–62.48 mg/100 g, and 6.2–7.3, respectively, as shown in Table 3. The combination with the highest bio‐fortified maize (60%) and lowest bean (15%) concentration had the highest provitamin A concentration of 1.6 μg RAE and the second highest overall acceptability of 7.2. In addition, the combination with the lowest maize (50%) and bean (15%) concentration resulted in a composite with the least provitamin A concentration of 1.0 μg RAE.

**TABLE 3:** Variation of provitamin A, iron, phytate content, and overall acceptability with the different composite flour combinations.

Run	A. O.M	B. Beans	C. Sesame	D. Soy	E. Sorghum	F. Wheat	Provitamin A, (μg RAE)/100 g	Iron, mg/100 g	Phytate, mg/100 g	O.A
1	52.6	20.0	5.0	12.4	5.0	5.0	1.1	6.39	62.5	6.6
2	56.0	19.0	5.0	10.0	5.0	5.0	1.4	6.23	58.0	6.9
3	56.0	19.0	5.0	10.0	5.0	5.0	1.4	6.25	57.9	6.7
4	50.0	18.4	10.0	11.6	5.0	5.0	1.0	6.15	56.1	6.5
5	50.0	20.0	7.3	12.6	5.0	5.0	1.0	6.34	60.4	6.2
6	50.0	20.0	5.1	14.9	5.0	5.0	1.0	6.36	60.4	6.4
7	53.6	17.1	7.1	12.1	5.0	5.0	1.2	5.95	54.6	6.5
8	53.6	20.0	6.3	10.0	5.0	5.0	1.2	6.40	60.6	6.7
9	55.5	17.0	5.0	12.5	5.0	5.0	1.4	5.92	54.3	6.8
10	54.3	15.7	5.0	15.0	5.0	5.0	1.2	5.79	53.8	6.7
11	60.0	15.0	5.0	10.0	5.0	5.0	1.6	5.69	53.0	7.2
12	57.2	15.0	7.8	10.0	5.0	5.0	1.4	5.67	53.2	7.1
13	50.0	17.9	7.1	15.0	5.0	5.0	1.0	6.09	55.4	6.7
14	52.5	15.0	9.9	12.6	5.0	5.0	1.1	5.68	53.0	6.7
15	53.6	17.1	7.1	12.1	5.0	5.0	1.2	5.96	54.1	6.9
16	53.6	17.1	7.1	12.1	5.0	5.0	1.2	5.95	54.9	6.8
17	53.6	17.1	7.1	12.1	5.0	5.0	1.2	5.94	54.8	7.1
18	50.0	17.9	7.1	15.0	5.0	5.0	1.0	6.07	55.1	6.4
19	54.3	15.7	5.0	15.0	5.0	5.0	1.2	5.78	53.9	6.9
20	53.6	17.1	7.1	12.1	5.0	5.0	1.2	5.97	54.2	6.7
21	54.6	15.4	10.0	10.0	5.0	5.0	1.3	5.71	53.8	6.8
22	51.6	20.0	8.4	10.0	5.0	5.0	1.1	6.37	60.3	6.6
23	54.6	15.4	10.0	10.0	5.0	5.0	1.3	5.74	53.9	6.5
24	52.3	15.1	7.6	15.0	5.0	5.0	1.1	5.71	53.8	6.6
25	50.0	18.4	10.0	11.6	5.0	5.0	1.0	6.16	56.5	6.5
26	51.6	20.0	8.4	10.0	5.0	5.0	1.1	6.35	61.7	6.3
27	54.8	15.0	7.5	12.7	5.0	5.0	1.3	5.65	53.6	6.9
28	53.6	17.1	7.1	12.1	5.0	5.0	1.2	5.96	54.8	7.0
29	50.0	15.0	10.0	15.0	5.0	5.0	1.0	5.66	53.7	6.4
30	57.70	15.00	5.25	12.05	5.00	5.00	1.45	5.62	53.67	7.3

Abbreviations: O.A, overall acceptability; O.M, orange maize.

### 3.2 Properties of the Composite Flour Obtained From Varying the Combinations of the Independent Variables

#### 3.2.1 Variation of Provitamin A Content With the Different Composite Flour Combinations

**FIGURE 1:**
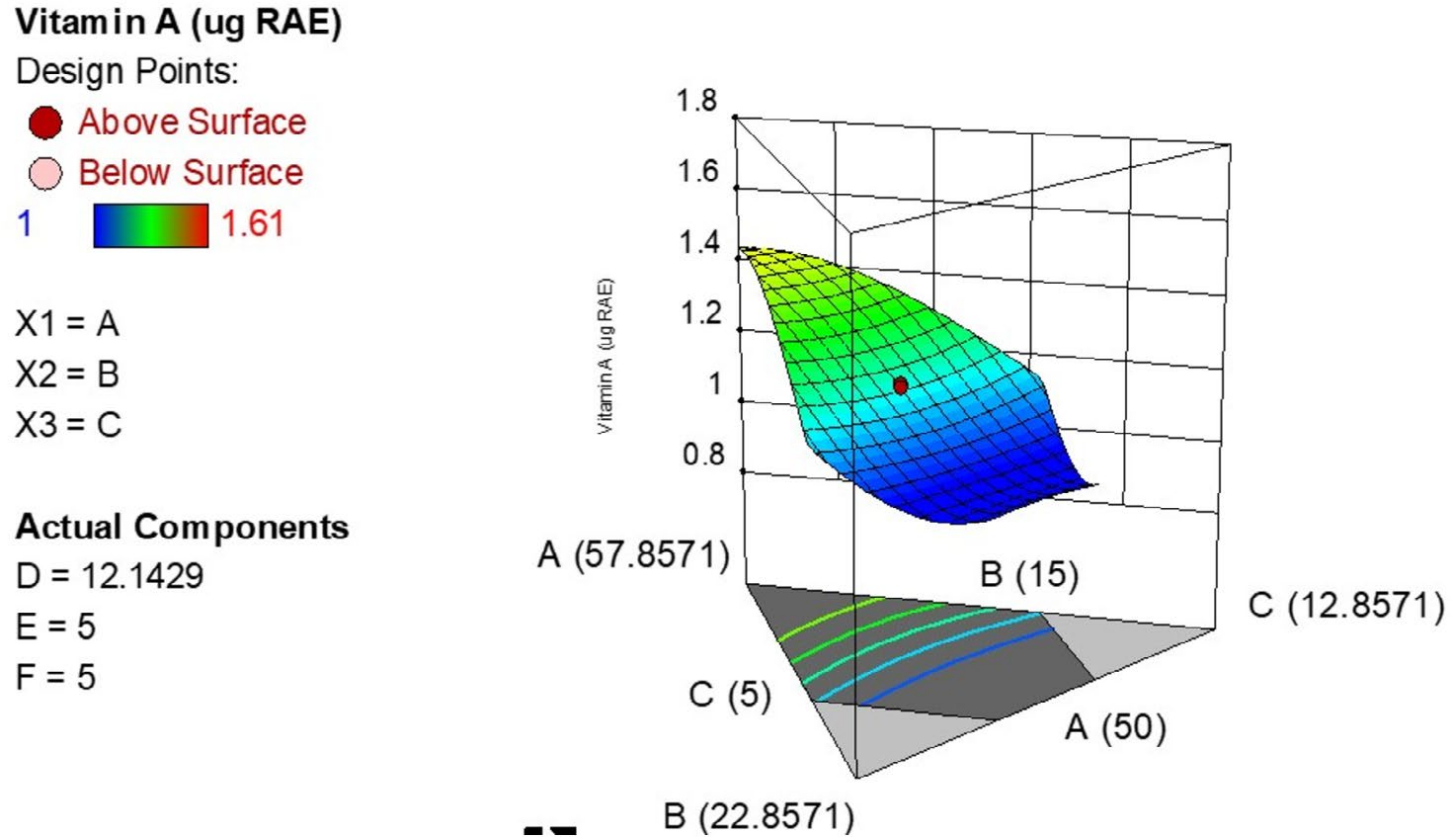
Effect of ingredient levels on the concentration of beta‐carotene (A is bio‐fortified maize, B is soy bean, C is sesame).

The beta‐carotene content of the composite flour ranged from 1.00 to 1.61 μg RAE/100 g. The predicted *R* ^2^ (0.9802) and the adjusted *R* ^2^ (0.9975) were adjacent (as shown in Table 4) showing close agreement between the experimental and predicted results. The model was significant and had a nonsignificant lack of fit at *p* < 0.05. The significant model terms were AB, AC, BC, BD, CD, ABC, ACD, BCD, AB (A‐B), ABCD, A^2^BC, A^2^CD. Equation (3) illustrates how the beta‐carotene varied with the independent variables (after removing insignificant terms) while Figure 1 is a 3D plot showing the variation of provitamin A with the ingredient levels.

(3) $$\begin{array}{c} \text{Beta carotene}\;\left(\mathrm{\mu g}\;\mathrm{RAE}/100\;g\right)=0.26A+29.35B+25.86C\\ \;+16.64D-0.62\mathrm{AB}-0.48\mathrm{AC}-2.30\mathrm{BC}-1.24\mathrm{BD}\\ \;-0.15\mathrm{CD}+0.05\mathrm{ABC}+-0.04\mathrm{ACD}+0.11\mathrm{BCD}\\ \;+\mathrm{AB}\;\left(A-B\right)-\text{ABCD}-A^{2}\mathrm{BC}+A^{2}\mathrm{CD}. \end{array}$$

where A is the orange maize, B is high iron beans, C is sesame, and D is soy bean.

**TABLE 4:** Regression model of relationship between independent variables and beta‐carotene.

Source	Sum squares	Degrees of freedom	Mean square	*F*‐value	*p*	
Model	0.7455	17	0.0439	671.27	< 0.0001	Significant
AB	0.0004	1	0.0004	6.71	0.0236	
AC	0.0007	1	0.0007	11.42	0.0055	
BC	0.0013	1	0.0013	20.43	0.0007	
BD	0.0008	1	0.0008	12.27	0.0044	
CD	0.0011	1	0.0011	17.25	0.0013	
ABC	0.0008	1	0.0008	12.30	0.0043	
ACD	0.0015	1	0.0015	23.47	0.0004	
BCD	0.0014	1	0.0014	21.21	0.0006	
AB (A‐B)	0.0012	1	0.0012	18.47	0.0010	
ABCD	0.0017	1	0.0017	26.57	0.0002	
A^2^BC	0.0005	1	0.0005	7.85	0.0160	
A^2^CD	0.0012	1	0.0012	17.83	0.0012	
Residual	0.0008	12	0.0001			
Lack of fit	6.049E‐07	1	6.049E‐07	0.0085	0.9282	Not significant
	**Value**					
*R* ^2^	0.9989					
*R* ^2^ adjusted	0.9802					
*R* ^2^ predicted	0.99775					

The predicted *R* ^2^ value of 0.9802 was in reasonable agreement with the adjusted *R* ^2^ value of 0.9975. The *R* ^2^ value is an indication of the level of responses that can be explained by the model (Gao et al. 2024). This implies that 98% and 99.8% of the responses could be explained by the model and, therefore, the model was significant in predicting the beta carotene content of the composite flour. Increasing the concentration of maize, beans, sesame, and soy had a positive linear effect on the beta carotene content of the composite flour. On the other hand, decreasing the concentration of maize in the composite flour while increasing other components had a negative effect on the beta carotene content. The beta carotene content of the composite generally increased with increase in the bio‐fortified maize concentration. Bio‐fortified maize is reported to have a beta carotene content as high as 15 μg/g (Menkir et al. 2018). This is higher than the beta carotene content of the other components of the composite. With reference to this study, bio‐fortified maize constituted the highest percentage of the composite at an inclusion level between 50% and 60%. Therefore, increasing the concentration of bio‐fortified maize ultimately increases the overall concentration of the beta carotenes in the composite flour and vice versa. Results from this study align with the findings of Alamu, Olatunde, et al. (2021); Alamu, Prisca, et al. (2021) who observed that varying concentrations of bio‐fortified maize and soy flour affected the beta carotene content in a composite flour. Specifically, decreasing the proportion of bio‐fortified maize while increasing soy flour led to a reduction in beta carotene content of the composite flour. The reduction in the beta carotene content can be attributed to the fact that soy contains less beta carotenes compared to bio‐fortified maize. In addition, Alamu, Olatunde, et al. (2021); Alamu, Prisca, et al. (2021) reported a higher provitamin A content for 100% pure bio‐fortified maize flour compared to composite flours containing bio‐fortified maize. The negative effect observed when the quantity of bio‐fortified maize flour is reduced could possibly result from a dilution effect which results when the quantities of other components which are low in beta carotene are added to the composite. Dilution effects have been reported elsewhere such as Tumwine et al. (2019) reported a decrease in the carbohydrate content of a composite flour due to the addition of skimmed milk and vegetables which were low in carbohydrate.

#### 3.2.2 Variation of Iron Content With the Different Composite Flour Combinations

**FIGURE 2:**
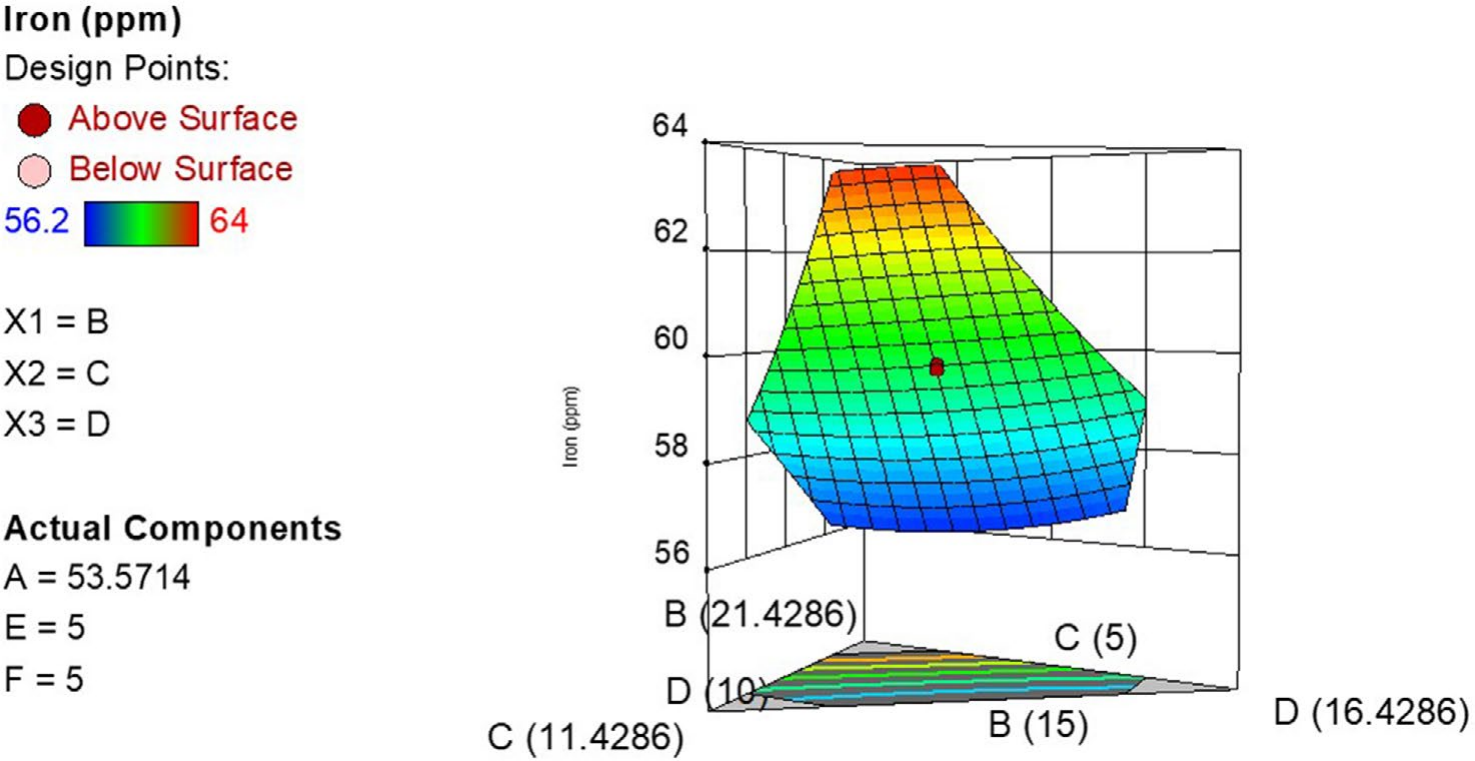
Effect of ingredient levels on iron concentration (B is iron‐rich beans, C is sesame, D is soy bean).

The iron content of the composite flour ranged from 5.62 to 6.39 mg/100 g. The predicted *R* ^2^ (0.9872) and the adjusted *R* ^2^ (0.9964) as shown in Table 5 were very close showing close agreement between the experimental and predicted results. The model was significant and had a nonsignificant lack of fit at *p* < 0.05. Equation (4) shows how iron content of composite flours varied with the independent variables (after removing insignificant terms) while Figure 2 is a 3D plot showing the variation of iron with the ingredient levels.

(4) $$\begin{array}{c} \text{Iron}\;\left(\mathrm{mg}/100\;g\right)=2.46A+14.41B+18.24C+28.52D\\ \;-0.29\mathrm{AC}-0.54\mathrm{AD}-0.43\mathrm{BC}-1.38\mathrm{BD}-1.66\mathrm{CD}\\ \;+0.02\mathrm{ABD}+0.02\mathrm{ACD}+0.03\mathrm{BCD} \end{array}$$

where A is the orange maize, B is high iron beans, C is sesame, and D is soy bean.

**TABLE 5:** Regression model of relationship between independent variables and iron.

Source	Sum of squares	Degrees of freedom	Mean square	*F*‐value	*p*	
Model	201.84	13	15.53	612.27	< 0.0001	Significant
Linear mixture	201.04	3	67.01	2642.66	< 0.0001	
AB	0.1041	1	0.1041	4.11	0.0597	
AC	0.1938	1	0.1938	7.64	0.0138	
AD	0.5590	1	0.5590	22.05	0.0002	
BC	0.1433	1	0.1433	5.65	0.0303	
BD	0.4490	1	0.4490	17.71	0.0007	
CD	0.5447	1	0.5447	21.48	0.0003	
ABC	0.0004	1	0.0004	0.0172	0.8973	
ABD	0.2533	1	0.2533	9.99	0.0061	
ACD	0.3153	1	0.3153	12.43	0.0028	
BCD	0.1830	1	0.1830	7.22	0.0162	
Residual	0.4057	16	0.0254			
Lack of fit	0.2357	5	0.0471	3.05	0.0572	Not significant
	**Value**					
*R* ^2^	0.9980					
*R* ^2^ adjusted	0.9964					
*R* ^2^ predicted	0.9872					

It was observed that increasing the concentration of maize, bean flour, sesame flour, and soy flour had a positive impact on the iron content of the composite flour. The iron content of the flour increased with the addition of bio‐fortified bean flour. This is because bio‐fortified beans are specifically bred for high iron content and therefore have a higher iron content compared to the other components in the composite flour (Beebe 2020; Glahn et al. 2020). Hence, as the bean flour concentration increases, the iron concentration also increases. This trend was similar to that observed in a study by Adoko et al. (2023) who found that the addition of a bio‐fortified orange fleshed sweet potato and iron‐rich beans to a composite flour consequently increased the iron concentration of the composite.

#### 3.2.3 Variation of Phytate Content With the Different Composite Flour Combinations

**FIGURE 3:**
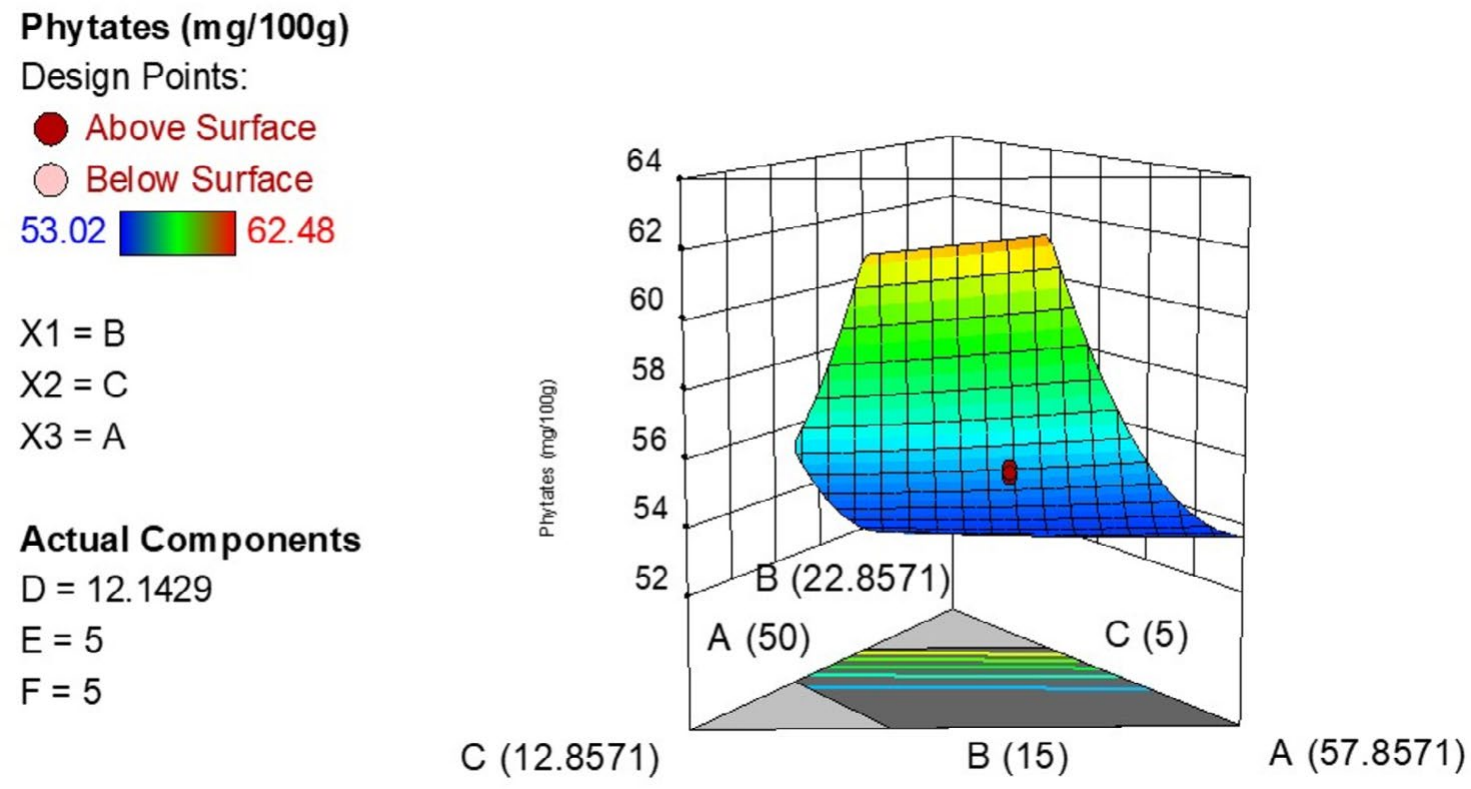
Effect of ingredient levels on the phytate concentration (A is bio‐fortified maize, B is iron‐rich beans, C is sesame).

The phytate content of the experimental runs ranged from 53.02 to 62.48 mg/100 g. The experimental and theoretical results were in close agreement, indicated by close proximity between predicted *R* ^2^ (0.9468) and adjusted *R* ^2^ (0.9723) values (Table 6). The model was significant and its lack of fit was not significant at *p* < 0.05. Equation (5) shows how phytate content varied with the independent variables (after removing nonsignificant terms) while Figure 3 is a 3D plot showing the variation of phytate content with the ingredient levels.

(5) $$\begin{array}{c} \text{Phytate content}\;\left(\mathrm{mg}/100\;g\right)=1.25A+23.47B+3.43C\\ \;+0.03D-0.36\mathrm{AB}-0.42\mathrm{BC}-0.38\mathrm{BD} \end{array}$$

where A is the orange maize, B is high iron beans, C is sesame, and D is soy bean.

**TABLE 6:** Regression model of relationship between independent variables and phytate content.

Source	Sum of squares	Degrees of freedom	Mean square	*F*‐value	*p*	
Model	237.11	9	26.35	114.01	< 0.0001	Significant
Linear mixture	207.04	3	69.01	298.65	< 0.0001	
AB	22.56	1	22.56	97.65	< 0.0001	
AC	0.0205	1	0.0205	0.0887	0.7689	
AD	0.3541	1	0.3541	1.53	0.2301	
BC	18.14	1	18.14	78.49	< 0.0001	
BD	17.30	1	17.30	74.86	< 0.0001	
CD	0.0817	1	0.0817	0.3535	0.5588	
Residual	4.62	20	0.2311			
Lack of fit	3.02	9	0.3357	2.31	0.0960	Not significant
	**Values**					
*R* ^2^	0.9809					
Adjusted *R* ^2^	0.9723					
Predicted *R* ^2^	0.9468					

Maize, beans, soy, and sesame flour had a positive linear effect on the phytate concentration of the composite flour. Though high iron bio‐fortified beans were used in this study, they have been found to have higher amounts of phytate compared to the conventional beans (Petry et al. 2014). Thus, increasing the concentration of beans increases the phytate concentration of the composite flour. Hoppler et al. (2014) correlated an increased iron content with an increased phytic acid in bio‐fortified beans. Because the bean variety utilized in the current study is characterized as having high iron and zinc content resulting from the bio‐fortification process, the increase in bean concentration will also increase the phytate concentration of the composite flour. In addition, soybeans and white sesame seeds have also been known to have elevated phytic acid concentrations and therefore increase the phytate content of the composite flour (Phescatcha et al. 2012). Although phytates have been scientifically proven to impair the absorption of Ca, Fe, and Zn thus affecting their bioavailability in humans, they are also associated with health benefits such as lowering lipid levels and performing some antioxidant and immune modulatory functions (Gibson et al. 2010). According to Nissar et al. (2017), phytate consumption should be reduced to ≤ 25 mg/100 g for better health outcomes hence a need for further studies on how to reduce the phytate content of foods. Because some bio‐fortified crops contribute to the antinutrient content of foods, there is a need to conduct extensive health studies before a cultivar is chosen for food production applications (Celmeli et al. 2018).

#### 3.2.4 Variation of Overall Acceptability of Composite Porridge With the Different Composite Flour Combinations

**FIGURE 4:**
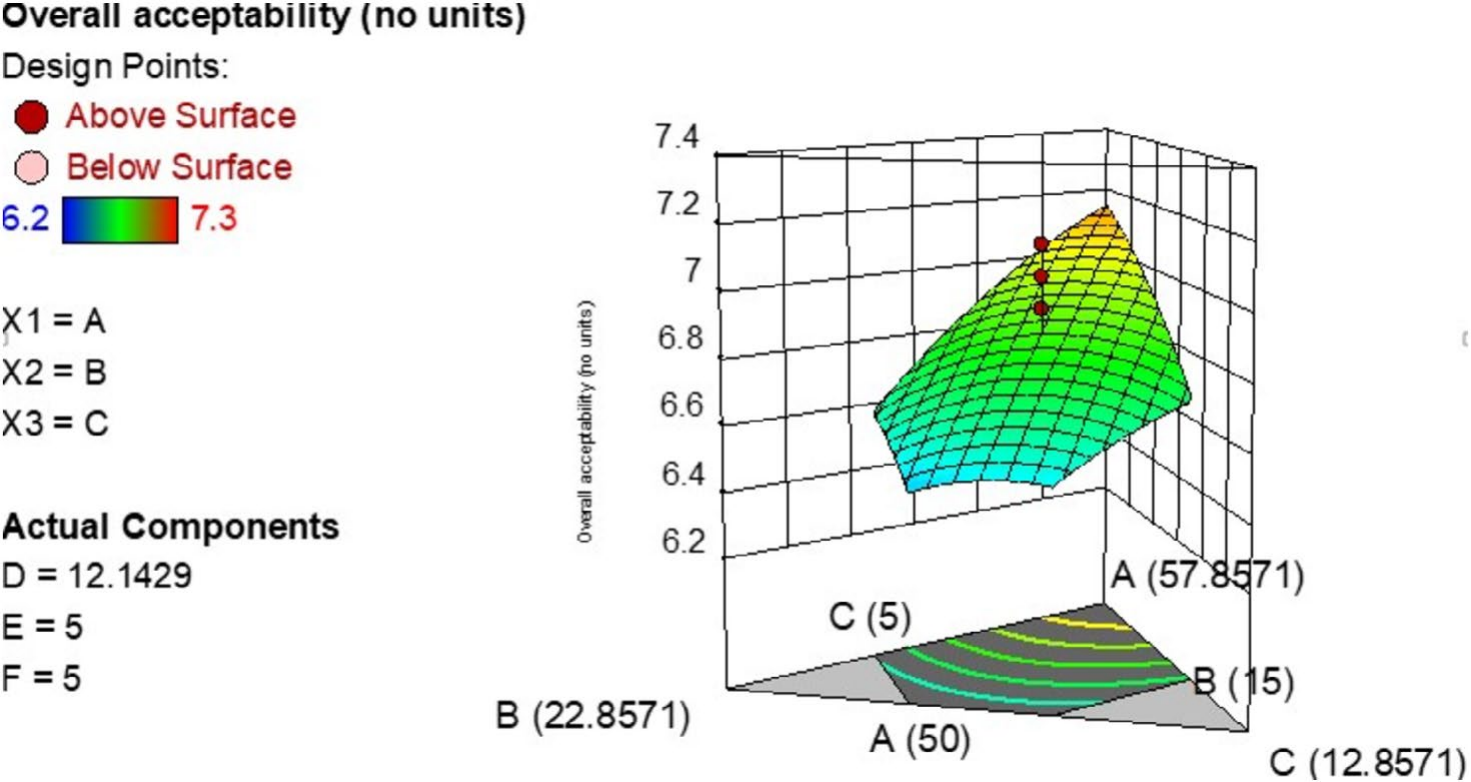
Effect of ingredient levels on overall acceptability (A is bio‐fortified maize, B is iron‐rich beans, C is sesame).

The overall acceptability of the experimental runs ranged from 6.2 to 7.3. The predicted *R* ^2^ was 0.7442 while the adjusted *R* ^2^ was 0.6016 as shown in Table 7. The model was significant and had an insignificant lack of fit at *p* < 0.05. Equation (6) shows how overall acceptability of composite porridges varied with the independent variables (after removing insignificant terms) while Figure 4 is a 3D plot showing the variation of overall acceptability with the ingredient levels.

(6) Overall acceptability=0.08A−0.6B−0.76C−0.68D

where A is the orange maize, B is high iron beans, C is sesame, and D is soybean.

**TABLE 7:** Regression model of relationship between independent variables and overall acceptability.

Source	Sum of squares	Degrees of freedom	Mean square	*F*‐value	*p*	
Model	1.50	9	0.1672	5.86	0.0005	Significant
Linear mixture	1.38	3	0.4598	16.13	< 0.0001	
AB	0.0142	1	0.0142	0.4996	0.4878	
AC	0.0143	1	0.0143	0.5027	0.4865	
AD	0.0197	1	0.0197	0.6927	0.4151	
BC	0.0577	1	0.0577	2.02	0.1703	
BD	0.0573	1	0.0573	2.01	0.1718	
CD	0.0473	1	0.0473	1.66	0.2124	
Residual	0.5701	20	0.0285			
Lack of fit	0.1618	9	0.0180	0.4842	0.8571	Not significant
	**Values**					
*R* ^2^	0.7252					
*R* ^2^ adjusted	0.6016					
*R* ^2^ predicted	0.7442					

Increasing bio‐fortified maize concentration had a positive linear effect on the overall acceptability of the flour while increasing bean, soy, and sesame flour had a negative linear effect on the overall acceptability. These results align with those from various studies which confirm sensory acceptability of some bio‐fortified foods (Alabi 2021; Beswa et al. 2020). Govender et al. (2019) furthermore hypothesized that combining provitamin A bio‐fortified foods with other commonly consumed plant food items could help in improving some undesirable sensory properties of the provitamin A bio‐fortified foods. The combination of provitamin A bio‐fortified maize with the other five plant‐based flours used in this study could have increased the overall sensory acceptability of the composite flour. On the other hand, the decrease in the overall sensory acceptability with increase in the concentration of beans is associated with beany flavors and a resulting off‐color which is deemed unacceptable by consumers.

### 3.3 Optimum Formulation of the Composite Flour

The best predicted optimal solution for the composite flour had the highest desirability of 0.74 as shown in Table 8. The composition of the optimized flour was maize at 57.89, beans at 17.11, sesame at 5.00, soy at 10, wheat and sorghum at 5 each. The optimal responses being 1.59 μg RAE of beta carotene, 5.97 mg/100 g of iron, 54.18 mg/100 g of phytic acid, and 7.07 numerical score of overall acceptability. Few studies have reported about optimization using provitamin A bio‐fortified maize in composite flours. The beta carotene value of 1.58 μg/g RAE in the optimized flour reported in this study is higher than that of 1.37 μg/g RAE reported for a composite flour made using a traditional maize variety, bambara groundnut and mango pulp (Bukuni et al. 2022). This higher value of beta carotenes is an indicator that the optimization done using bio‐fortified provitamin A rich maize increased the beta carotene content of the composite flour. On the other hand, a study by Adegunwa et al. (2020) showed a higher beta carotene content ranging from 1.99 to 2.65 μg/g RAE for a composite flour containing soy and bio‐fortified maize. When compared to the current study, the lower values for the beta carotene in the current study could be explained by the fact that the bio‐fortified maize grains used in the formulation were from a previous harvest. Beta carotenoids in vitamin A bio‐fortified maize have been found to degrade during the postharvest period (Taleon et al. 2017). De Moura et al. (2015) noted that the highly unsaturated structure of carotenoids makes them susceptible to post harvest degradation by heat, oxygen and light. The iron value of 6.00 mg/100 g in the optimized flour was higher than that of 3.83 mg/100 g reported by Kambabazi et al. (2022) for a bean‐based complimentary food made using conventional beans. The addition of bio‐fortified beans, therefore, has the potential to improve the iron content of complementary foods prepared for children under the age of 5 years. The phytate content of 54.18 mg/100 g was higher than that reported for a composite flour containing non‐bio‐fortified maize (0.11 mg/100 g) (Bukuni et al. 2022). Phytates have been commonly reported to be present in cereals, pulses, nuts and seeds (López‐Moreno et al. 2022). Further still, bio‐fortified beans have been found to have higher amounts of phytate compared to the conventional beans (Petry et al. 2014). Though phytates have been known for their negative role in reducing iron absorption, they also play various positive roles such as in calcification, lowering blood glucose and lipids (Gemede 2014). They have also been found to have anti‐oxidative and anticancer genic properties (Schlemmer et al. 2009).

**TABLE 8:** Possible solutions of the optimized flour generated by design expert.

No.	Orange maize	Bean	Sesame	Soy beans	Sorghum	Wheat	Provitamin A	Iron	Phytate	O.A	DF
1	57.89	17.11	5.00	10.00	5.00	5.00	1.59	59.65	54.18	7.07	0.74^a^
2	57.21	17.57	5.19	10.02	5.00	5.00	1.55	60.25	54.90	7.00	0.72
3	57.27	17.25	5.48	10.00	5.00	5.00	1.57	59.72	54.39	7.02	0.72
4	56.77	17.49	5.56	10.19	5.00	5.00	1.52	60.02	54.80	6.99	0.70

Abbreviations: DF, desirability function; O.A, overall acceptability.

^a^ Selected optimized formulation.

#### 3.3.1 Validation of Optimum Parameters

Table 9 shows that there was no significant difference (*p* ≤ 0.05) between the predicted values under optimized conditions and the observed values for all the response variables. This indicated the suitability of the models in optimizing the formulation of the composite flour.

**TABLE 9:** Summary of the predicated and observed values of reponses.

	Provitamin A (μg RAE)	Iron (mg/100 g)	Phytate (mg/100 g)	Overall acceptability
Predicted	1.59 ± 0.00^a^	5.97 ± 0.00^a^	54.18 ± 0.00^a^	7.07 ± 0.00^a^
Observed	1.58 ± 0.04^a^	6.00 ± 0.01^a^	54.20 ± 0.15^a^	7.10 ± 0.21^a^

^a^ Values with the same superscript are not statistically different (*P*< 0.05).

## 4 Conclusion

Results from this study showed that the addition of provitamin A bio‐fortified maize into an iron‐rich bean‐based composite flour improved the vitamin A content, iron content, and overall acceptability of the composite flour but with an increment in the phytate content of the composite flour when compared with results from other studies. There is therefore a need for further studies on strategies that can be employed to reduce anti‐nutritional levels to acceptable levels. Although the provitamin A and iron content of the optimized complementary flour were not as high as those reported in similar studies involving bio‐fortified crops, they were higher than those reported in non‐bio‐fortified maize and bean composite flours. Bio‐fortified maize and beans should, therefore, be made a common component of complementary foods prepared with the aim of alleviating vitamin A and iron deficiency among children under the age of 5 years. For a better balance of essential amino acids, the use of milk instead of water during porridge preparations is recommended. There is a need for further research to evaluate how the addition of provitamin A bio‐fortified maize to the iron‐rich bean‐based composite flour affects the nutrient composition, functional properties, and sensory attributes of the composite flour.

## Author Contributions

**Amos Asiimwe:** conceptualization (equal), data curation (equal), formal analysis (equal), investigation (equal), methodology (equal), software (equal), validation (equal), visualization (equal), writing – original draft (lead), writing – review and editing (lead). **Juliana Nambwayo:** conceptualization (equal), data curation (equal), formal analysis (equal), investigation (equal), methodology (equal), writing – original draft (equal), writing – review and editing (equal). **Boniface Brian Odong:** writing – review and editing (equal). **Robert Fungo:** conceptualization (equal), funding acquisition (equal), methodology (equal), project administration (equal), supervision (equal), writing – original draft (equal), writing – review and editing (equal). **Ivan Muzira Mukisa:** supervision (equal), writing – original draft (equal), writing – review and editing (equal).

## Data Availability

The data that support the findings of this study are available from the corresponding author upon reasonable request.
